# A Novel Intravital Method to Evaluate Cerebral Vasospasm in Rat Models of Subarachnoid Hemorrhage: A Study with Synchrotron Radiation Angiography

**DOI:** 10.1371/journal.pone.0033366

**Published:** 2012-03-12

**Authors:** Jun Cai, Yuhao Sun, Falei Yuan, Lujia Chen, Chuan He, Yuhai Bao, Zuoquan Chen, Meiqing Lou, Weiliang Xia, Guo-Yuan Yang, Feng Ling

**Affiliations:** 1 Department of Neurosurgery, Shanghai Tenth People's Hospital, Tongji University School of Medicine, Shanghai, China; 2 Department of Neurosurgery, Xuanwu Hospital, Capital Medical University, Beijing, China; 3 Neuroscience and Neuroengineering Center, Med-X Research Institute, Shanghai Jiao Tong University, Shanghai, China; 4 Department of Neurosurgery, Ruijin Hospital, School of Medicine, Shanghai Jiao Tong University, Shanghai, China; University of Padova, Italy

## Abstract

Precise *in vivo* evaluation of cerebral vasospasm caused by subarachnoid hemorrhage has remained a critical but unsolved issue in experimental small animal models. In this study, we used synchrotron radiation angiography to study the vasospasm of anterior circulation arteries in two subarachnoid hemorrhage models in rats. Synchrotron radiation angiography, laser Doppler flowmetry-cerebral blood flow measurement, [^125^I]*N*-isopropyl-*p*-iodoamphetamine cerebral blood flow measurement and terminal examinations were applied to evaluate the changes of anterior circulation arteries in two subarachnoid hemorrhage models made by blood injection into cisterna magna and prechiasmatic cistern. Using synchrotron radiation angiography technique, we detected cerebral vasospasm in subarachnoid hemorrhage rats compared to the controls (*p*<0.05). We also identified two interesting findings: 1) both middle cerebral artery and anterior cerebral artery shrunk the most at day 3 after subarachnoid hemorrhage; 2) the diameter of anterior cerebral artery in the prechiasmatic cistern injection group was smaller than that in the cisterna magna injection group (*p*<0.05), but not for middle cerebral artery. We concluded that synchrotron radiation angiography provided a novel technique, which could directly evaluate cerebral vasospasm in small animal experimental subarachnoid hemorrhage models. The courses of vasospasm in these two injection models are similar; however, the model produced by prechiasmatic cistern injection is more suitable for study of anterior circulation vasospasm.

## Introduction

Subarachnoid hemorrhage (SAH) is a vital clinical syndrome, nearly 80% of which is caused by the rupture of cerebral aneurysm. Approximately 10 in 100,000 people experience aneurysmal SAH every year, in which, about 40% die and 30% of the survivors suffer from morbidity [1]. Severe cerebral vasospasm (CV) is one of the major causes of mortality and morbidity in aneurysmal SAH [2]–[4]. Therefore, the prevention of refractory CV is always a primary concern in experimental and clinical studies.

To explore the mechanisms of experimental SAH pathophysiology, various approaches were tested on animal models; however, most of these methods were histological analyses with few *in vivo* interpretations. The *in vivo* evaluative tools of CV include laser Doppler flowmetry (LDF)-cerebral blood flow (CBF) measurement, computed tomography angiography (CTA), magnetic resonance and digital subtraction angiography (DSA) [5]–[9]. Because of limited resolution or indirection, these methods could not be widely used to detect and evaluate CV in experimental SAH models. Synchrotron radiation (SR) has been regarded as a unique tool to visualize pathophysiologic changes of small arteries [10]. SR is a method that uses two monochromatic X-ray beams to closely bracket the K-edge of iodine (33.164 keV), which provides two simultaneous images with one above and the other below the K-edge. Logarithmic subtraction of the images provided by these beams results in an image that enhances signals arising from attenuation by the photoelectric effect of iodine and suppresses signals arising from attenuation by soft tissue and bone [11]. Therefore, SR seems to be a promising tool to detect and evaluate vasospasm in animal SAH models *in vivo*.

In experimental SAH, animal models such as rabbits, dogs, cats, pigs, primates, rats and mice are used [12]–[23]. Among them, rats were the most widely used [18], [24]. SAH rat models which are deemed to induce vasospasm of anterior circulation arteries are internal carotid artery perforation and prechiasmatic cistern injection [25]–[27]. Injection of autologous blood into cisterna magna is the most widely used method because it is simple operated with low mortality [28]–[30]. However, whether cisterna magna injection of rats produces pronounced vasospasm of anterior circulation arteries, like prechiasmatic cistern injection does, remains unknown. It is due to the blood is mainly distributed into the posterior cranial fossa and the spinal canal [27].

In the present study, we aim to assay whether synchrotron radiation angiography (SRA) can detect and evaluate cerebral vasospasm in two SAH models. At the meantime, we would like to compare the severity of vasospasm of anterior circulation arteries produced by these two models.

## Materials and Methods

### Experimental animal groups

Animal procedures were carried out according to a protocol approved by the Institutional Animal Care and Use Committee (IACUC) at Shanghai Jiao Tong University, Shanghai, China. Two hundreds and six adult male Sprague-Dawley rats (Slac Laboratory Animal Co., Shanghai, China) weighing 350–400 g were used in this study. The rats were housed in the animal room at 22–24°C with 12-hour light/dark circle and free access to food and water. They were randomized into five groups, which were G0 (control group, n = 18), G1 (saline-injected into cisterna magna, n = 39), G2 (saline-injected into prechiasmatic cistern, n = 38), G3 (blood-injected into cisterna magna, n = 57) and G4 (blood-injected into prechiasmatic cistern, n = 54).

### Induction of experimental SAH

The rats were anaesthetized by intraperitoneal injection of 100 mg/Kg ketamine (Gu-Tian Ltd., Fujian, China) and 10 mg/Kg xylazine (Sigma-Aldrich Co., St Louis, MO, USA). After anesthesia, non-heparin blood was extracted into a 1 ml syringe from left femoral artery. A heating pad (RWD Life Science Co.; Shenzhen, China) was used to maintain animal temperature at 37.0±0.5°C. Rats of G1 and G3 groups were fixed in a stereotactic frame equipped with a rat mouth holder (RWD Life Science Co.; Shenzhen, China) and placed in a prone position as described previously [28], [31], [32]. Under an operating microscope (Leica Co., Heerbrugg, Switzerland), the nuchal muscle layers were divided in the midline, retracted laterally to expose the lamina of atlas and the atlanto-occipital membrane. A small midline burr hole (less than 1 mm in diameter) was made just rostrally to the interparietal-occiptal suture using a high-speed drill (Fine Science Tool Inc., Foster City, CA, USA) and care was taken not to open the cisterna magna. While the cisterna magna was being viewed under high magnification through the transparent dura mater, a PE-10 catheter attached to the 1 ml syringe was introduced along the inner table of the occipital bone into the cisterna magna at an angle of about 60 degrees with the top of the calvarium, until the catheter tip was visible within cisterna magna. Under manual manipulation, 0.1 ml cerebral-spinal fluid (CSF) was gently aspirated, followed by injection of 0.2 ml saline (G1) or 0.2 ml blood (G3) during a period of approximate 3 minutes. Then, the animals were tilted 30 degrees with the head down for 30 minutes. After 24 hours of first operation, the foregoing procedure was repeated with injection of 0.2 ml saline (G1) or 0.2 ml blood (G3).

Rats in G2 and G4 groups were fixed in a stereotactic frame and placed in a prone position [27]. A midline incision on the calvarium was made, then, a burr hole at 7.5 mm anterior to bregma and 0.5 mm right away from midline was drilled. A 27-gauge needle attached to a 1 ml syringe with non-heparin blood was inserted into the hole and tilted 30 degrees in the sagittal plane, and the syringe was connected to a microinjecting pump (World Precision Instruments Inc., Sarasota, FL, USA); the needle was lowered 2–3 mm until the tip reached the base of the skull, then withdrew, if the needle was into prechiasmatic cistern, transparent CSF was back-flowed in the syringe. When the needle was placed into prechiasmatic cistern, 0.2 ml saline (G2) or 0.2 ml blood (G4) at a speed of 100 µl/min was injected mechanically.

Animals were returned to their cages after awakening until SRA or euthanization.

### Intracranial pressure (ICP) measurement

ICP was measured continuously during SAH in G1, G2, G3 and G4 groups using a physiological monitor (PowerLab, ADinstrument Co., Australia). According to Barth [33], before induction of SAH, a burr hole right side to the midline was made rostrally to the interparietal-occiptal suture and placed a special bulb into cisterna magna, which was connected to a pressure transducer (PowerLab, ADinstrument Co., Australia) through a PE-50 tube. The ICP values of 5 minutes before SAH and 5, 10, 15, 20, 25, 30, 35, 40, 45, 50, 55, 60 minutes after SAH were recorded.

### Mortality and neurological deficit assessment

Forty-eight hours after SAH induction, the mortality was calculated. The rats from G1, G2, G3 and G4 groups were subject to neurological status evaluation at day 3 by an observer blindly using a neurological scoring system [34].

### Surface cerebral blood flow (CBF) measurement via a laser Doppler flowmetry (LDF)

**Figure 1:**
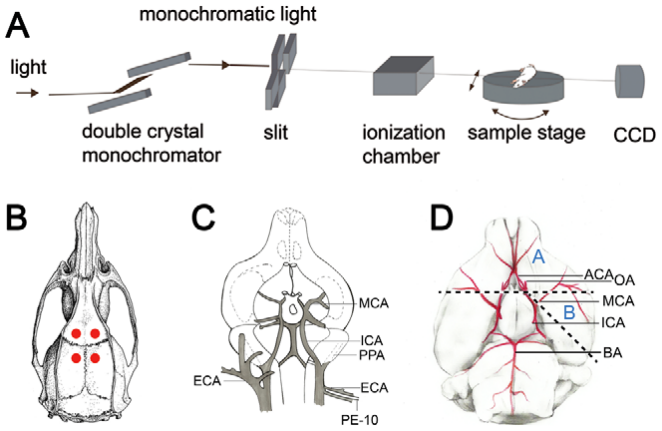
A schematic presentation of BL13W-Line Station of Shanghai Synchrotron Radiation Facility (A). The synchrotron beam is monochromatized and penetrated the animal on the sample stage, then received by a CCD camera. A picture (**B**) illustrates four burr holes on the calvarium used to measure surface CBF with laser Doppler flowmetry. A schematic drawing of catheterization with major blood vessels labeled (**C**). A PE-10 tube is inserted into ECA and advanced upon to the bifurcation of ECA and ICA. A Cartoon (**D**) illustrates the circle of Willis in a rat brain. Dotted lines indicate the locations of brain cut. Sample A is prepared for ACA and sample B is prepared for MCA. ACA: anterior cerebral artery, BA: basilar artery, CCA: common carotid artery, ECA: external carotid artery, ICA: internal carotid artery, MCA: middle cerebral artery, OA: olfactory artery, PPA: pterygopalatine artery.

Surface CBF measurement was performed via a LDF (Moor Instruments Co., Axminster, UK). Four burr holes were drilled at 2.5 mm bilateral/1.0 mm posterior to bregma or 2.5 mm bilateral/3.5 mm anterior to bregma (**Figure 1B**). The holes were 2 mm in diameter. LDF was employed to detect the surface CBF supplied by bilateral middle cerebral artery (MCA) or anterior cerebral artery (ACA) before operation and 30 minutes, 1 to 7 days after SAH. Values before operation were used as baseline. CBF in G1 and G3 groups had been measured before the first operation was used as baseline. Rats were anaesthetized with ketamine and xylazine following foregoing protocols. The same cortex was measured three times and averaged to give the value.

### [^125^I]*N*-isopropyl-*p*-iodoamphetamine ([^125^I]-IMP)-CBF measurement

The *N*-isopropyl-*p*-amphetamine (IMP) was purchased from Sigma-Aldrich Co. (Sigma-Aldrich Co., St Louis, MO, USA); the raw material [^125^I] was purchased from Nuclear Power Institute of China (Sichuan, China); the [^125^I]-IMP was synthesized by Institute of Radiation Medicine, Fudan University (Shanghai, China). 1, 3, 5 and 7 days after SAH, rats in G0, G1, G2, G3 and G4 groups were injected 320 kBq [^125^I]-IMP through a tail vein. Rats were decapitated 30 minutes after [^125^I]-IMP injection followed by quick brain removals. Ci-IMP(t) of the cerebrum, the tissue concentration of the ^125^I, was measured with the γ-counter (SN-695, Shanghai Hesuo Rihuan Photoelectric Instrument Co., Shanghai, China). According to a simplified CBF measurement, CBF of each animal was quantitated with differential uptake ratio (DUR), and DUR was calculated by the equation: DUR = Ci−IMP(t)/D×BW [35]; where D and BW were injected dose (cpm) and body weight (gram).

### Procedures of synchrotron radiation angiography (SRA)

Animals in G0, G1, G2, G3 and G4 groups were transported to Shanghai Synchrotron Radiation Facility (SSRF) for SRA at 1, 3, 5 and 7 days after SAH. SSRF is a newly established third-generation SR light source with an accelerator of 3.5 GeV [36]–[38]. The synchrotron beam was monochromatized by two pieces of silicon crystal, which were placed in front of the imaging hutch (**Figure 1A**) [39]. The parameter of imaging: average beam current was 200 mA, X-ray energy was 33.5 keV, distance between a rat and a CCD camera with resolution of 13 µm (Photonic-Science Co., East Sussex, UK) was 65 cm, a field of view (FOV) was 45 mm (H)×2.5 mm (V). The image of hemispheric angiography of a normal rat was combined with four and five slices.

Animals were anaesthetized by ketamine 100 mg/Kg and xylazine 10 mg/Kg ip. After anesthesia, rats were placed in a supine position, a midline incision was made on the neck under an operating microscope; left common carotid artery (CCA), external carotid artery (ECA) and internal carotid artery (ICA) were isolated. A PE-10 tube was inserted into ECA and advanced upon to the bifurcation of ECA and ICA (**Figure 1C**). The PE-10 tube was connected to a PE-50 tube. After catheterization, rats were placed on their sides and vertical to the beam and CCD (**Figure 1A**) [39]. Nonionic iodine, Omnipaque (GE Healthcare Co., Buckinghamshire, UK) was injected as a contrast agent through the PE-50 catheter, the injection speed was 3 ml/min and injecting volume was 150 µl. SRA images were taken immediately after iodine injection. The diameters of ICA, MCA and ACA were measured using Image-Pro Plus program 6.0 (Media Cybernetics Inc., Bethesda, Maryland, USA). ICA was measured at bifurcation of ICA and pterygopalatine artery (PPA); MCA was measured at bifurcation of MCA and the circle of Willis; and ACA was measured at bifurcation of ACA and olfactory artery (OA) by an observer blindly.

### Examinations of histology and micro-XCT

Rats in G0, G1, G2, G3 and G4 groups were euthanized for morphological examination at 1, 3, 5, and 7 days of SAH. Animals were incised to expose the right atrium and perfused through the left cardiac ventricle with 50 ml physiological saline, followed by 50 ml 4% paraformaldehyde. The brains were removed and fixed in 4% paraformaldehyde for 24 hours. Under operating microscope, brain samples vertical to ACA or MCA were cut (**Figure 1D**). Brain tissues were embedded in paraffin with automatic embedding machine (Leica Co., Nussloch, Germany), and sections in 5 µm thickness were made by a paraffin slicing machine (Leica Co., Nussloch, Germany). After 24–48 hours, sections were deparaffinized in xylene and rehydrated through a decreasing gradient of ethanol solutions. Slides were stained with hematoxylin and eosin, mounted and viewed under a light microscope (Leica Co., Wetzlar, Germany). The diameter of MCA at 100 µm away to the bifurcation, and the diameter of ACA at 200 µm away to the bifurcation were measured by an observer blindly using a Image J software (NIH Program, Bethesda, Maryland, USA).

Rats from control group (G0) and SAH group (G4) were subject to micro-XCT scan. After perfusion of 50 ml physiological saline, Microfil (50 ml; Flow Tech, MA, USA) was perfused through left cardiac ventricle. Four hours after perfusion, the brains were removed and fixed in 4% paraformaldehyde for 24 hours. The brains were examined with a micro-XCT scanner (Xradia, CA, USA).

### Statistic analysis

Statistical analysis was performed with SPSS 16.0 (SPSS Inc, Chicago, IL, USA). Data were presented as means±SD. Values of LDF-CBF and [^125^I]-IMP CBF, diameters of ICA, MCA and ACA (measured through SRA images or histological sections), as well as neurological scores were calculated with one-way analysis of variance (ANOVA) or Kruskal-Wallis *H* test. Correlations of MCA and ACA diameter (measured through SRA images) and values of specific surface CBF were calculated using a Pearson correlation. A *p*<0.05 was considered significant.

## Results

### ICP and CBF changes

**Figure 2:**
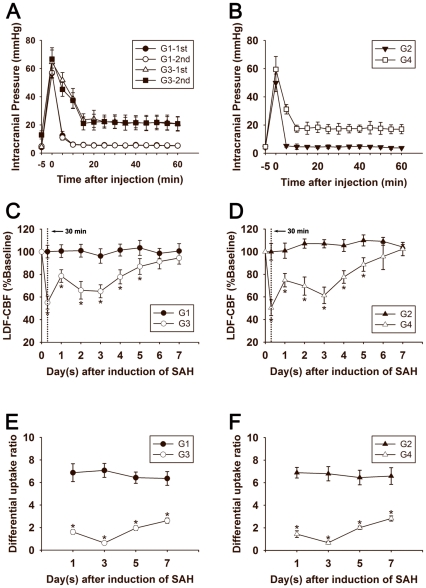
Line graphs (A and B) show the changes of ICP within 60 minutes following operation in G1, G2, G3 and G4 groups. Data are means±SD, n = 5 in each group. G1 group: cisterna magna saline injection. G2 group: prechiasmatic cistern saline injection. G3 group: cisterna magna blood injection. G4 group: prechiasmatic cistern blood injection. ICP: intracranial pressure, SAH: subarachnoid hemorrhage. Line graphs (**C** and **D**) show the surface CBF changes following 7 days after operation. Data are means±SD, n = 8 in each group. *, p<0.05, G1 vs. G3, G2 vs. G4. CBF: cerebral blood flow. Line graphs (**E** and **F**) show the regional CBF (DUR) changes using isotope [^125^I]-IMP following 1, 3, 5, and 7 days after operation. Data are means±SD, n = 5 in each group. *, *p*<0.05, G1 vs G3, G2 vs G4. DUR: differential uptake ratio, [125I]-IMP: [^125^I]*N*-isopropyl-*p*-iodoamphetamine.

The baseline ICP was approximately 5 mm Hg in G1, G2, G3 and G4 groups. Immediately after blood or saline injection, ICP rapidly increased to 56.9±3.3 (G1), 49.9±6.2 (G2), 65.6±8.0 (G3) and 59.5±9.2 (G4). ICP in G1 and G2 groups reduced to normal level within 15 minutes, while ICP in G3 and G4 groups remained at a higher level for at least 60 minutes (**Figure 2A** and **B**).

The surface CBF in G3 and G4 groups reduced to about 50% of baseline at 30 minutes after SAH; their CBF remained in a low level of baseline (≈70%) at day 2 and 3, and gradually returned to the normal at 7 days. There was no change of the surface CBF in G1 and G2 groups. There were significant differences of the surface CBF in G1 and G3, G2 and G4 groups (*p*<0.05, **Figure 2C** and **D**). Regional CBF of brain tissues of cerebrum was quantified with DUR. The DUR value in G0, G1 and G2 groups were 6.9±1.0, 6.7±1.1 and 6.7±0.9. There was no significant difference of DUR in G0, G1 and G2 groups (*p*>0.05). The DUR of G1 and G3 groups at day 1, 3, 5 and 7 were 6.7±1.7, 7.3±1.2, 6.6±0.8, 6.3±0.6 (G1) and 1.6±0.2, 0.6±0.2, 2.0±0.2, 3.1±0.3 (G3) respectively; the DUR of G2 and G4 groups at each time point were 7.2±1.2, 6.8±1.1, 6.5±0.6, 6.5±0.8 (G2) and 1.4±0.3, 0.7±0.1, 2.0±0.1, 3.2±0.2 (G4) respectively. There were significant differences of DUR in G1 and G3, G2 and G4 groups (*p*<0.05, **Figure 2E** and **F**).

### Mortality and neurological deficit after SAH induction

**Figure 3:**
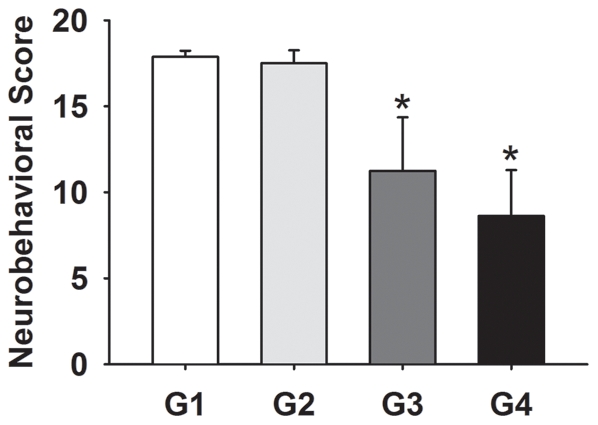
Bar graphs display the neurobehavioral scores of G1, G2, G3 and G4 groups at day 3 after operation. Data are means±SD, n = 8 in each group. *, *p*<0.05, G1 vs G3, G2 vs G4.

Forty-eight hours after SAH, mortality rates varied among the four groups with 1/39 (2.6%) in G1 group, 0/38 (0%) in G2 group, 13/57 (22.8%) in G3 group and 7/54 (13.0%) in G4 group. Neurological scores in G1, G2, G3 and G4 groups were 17.9±0.4, 17.5±0.8, 11.3±3.1 and 8.6±2.7 respectively at day 3 after SAH. The scores in G1 and G3 group, G2 and G4 groups had significant differences (*p*<0.05). Although the scores in G3 group were higher than those in G4 group, there was no significant difference between G3 and G4 (**Figure 3**).

### Measurement of cerebral arteries using SRA

**Figure 4:**
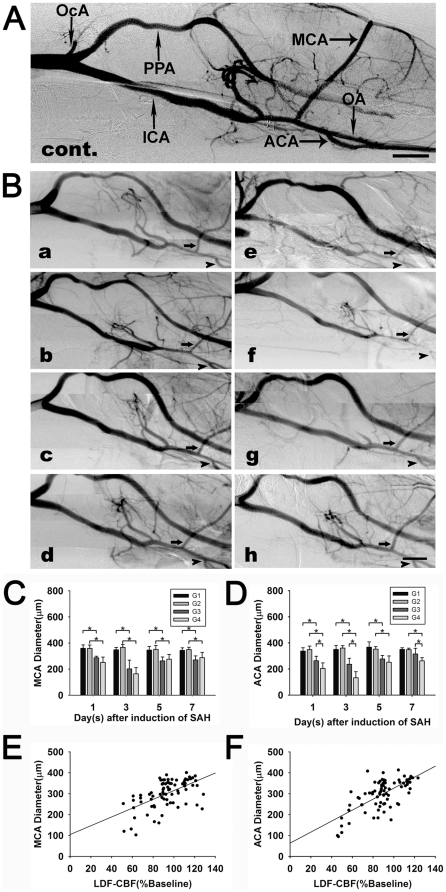
A representative SRA image of rat in control group (A). SRA imaging (**B**) at day 1 (a, e), 3 (b, f), 5 (c, g) and 7 (d, h) following SAH in cisterna magna blood injection (a, b, c, d) and prechiasmatic cistern blood injection group (e, f, g, h). Arrows indicate the vasospastic MCA; arrowheads indicate the vasospastic ACA. Scale bar = 2 mm. Bar graphs (**C** and **D**) show the MCA and ACA diameter in G1, G2, G3 and G4 groups at each time point after operation. Data are mean±SD. n = 5 in each group. *, *p*<0.05, G1 vs. G3, G2 vs. G4, G3 vs. G4. Correlation maps (**E** and **F**) display positive correlations between MCA (E), ACA (F) diameter (measured in SRA images) and the specific surface CBF in control and SAH groups. n = 5 in each group.

To directly evaluate CV *in vivo*, we performed SRA in all groups (**Figure 4A** and **B**). The cerebral vascular morphology of SRA imaging in G1 and G2 groups were similar to those in G0 group. ICA, MCA and ACA diameter in the normal control (G0) were 608±62, 359±19 and 347±28 µm respectively. It seemed that ICA, MCA and ACA diameter among G0, G1 and G2 groups had no significant difference (*p*>0.05). ICA diameter in G1, G2, G3 and G4 groups at 1, 3, 5 and 7 days of SAH were similar (*p*>0.05). MCA and ACA diameter in G3 and G4 groups were smaller than those in G1 and G2 groups respectively (*p*<0.05). The smallest MCA and ACA diameter in G3 and G4 groups were at day 3, which were 202±67 and 236±46 µm (G3), 164±48 and 133±47 µm (G4). ACA diameter in G4 group were smaller than those in G3 group (*p*<0.05). MCA and ACA diameter in G1, G2, G3 and G4 groups were shown in **Figure 4C** and **D**. MCA and ACA diameter were positively correlated with specific surface CBF (r = 0.576 and 0.678, *p*<0.05) (**Figure 4E** and **F**).

### Terminal examinations of cerebral arteries

**Figure 5:**
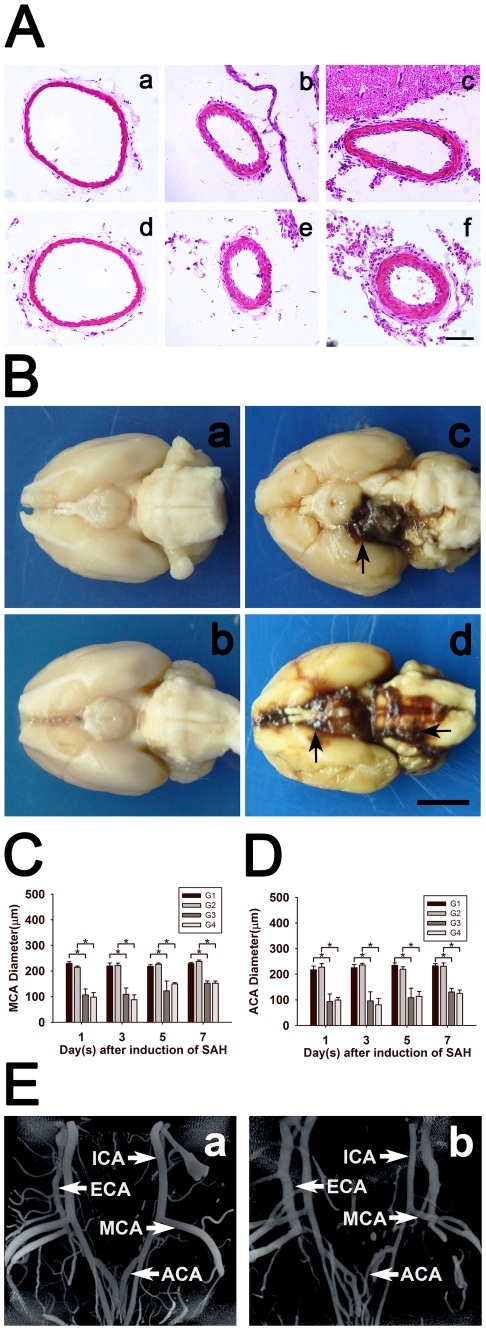
Photomicrographs (A) show HE staining of ACA (a, b, c) and MCA (d, e, f) following SAH in control (a, e), cisterna magna blood injection (b, e) and prechiasmatic cistern blood injection groups (c, f). Scale bar = 50 µm. Macroscopic images of brain samples after saline perfusion and paraformaldehyde fixation (**B**). Brains are obtained from G1 (a), G2 (b), G3 (c) and G4 (d) groups, respectively. Arrows indicate the blood clot at the basal subarachnoid space. Scale bar = 2 cm. Bar graphs (**C** and **D**) summarize the MCA and ACA diameter in G1, G2, G3, and G4 groups at each time point after operation. Data are means±SD. n = 5 in each group. *, *p*<0.05, G1 vs. G3, G2 vs. G4. Three dimensional photomicrographs (**E**) show major cerebral arteries of the Circle of Willis in control (a) and SAH rats (b) using micro-XCT imaging technique.

To compare results from SRA to the pathology, brains were examined. Extensive SAH in ventral surface were observed, especially around the circle of Willis region. The blood volume injected into cisterna magna or prechiasmatic cistern could result in the formation of clots around the Willis Circle as showed in **Figure 5B**. Histologically, MCA and ACA diameter in G0 group were 225±13 and 219±17 µm. There were no significant differences in MCA and ACA diameter among G0, G1 and G2 groups (*p*>0.05). MCA and ACA diameter at 1, 3, 5 and 7 days of SAH were 93±30, 96±35, 108±37, 130±14 (MCA) and 107±24, 109±24, 123±38, 153±9 (ACA) in G3 group, and 99±10, 80±25, 113±19, 125±13 (MCA) and 98±18, 88±19, 150±11, 152±9 (ACA) in G4 group, which were smaller than those in G1 and G2 groups respectively (*p*<0.05). Vasospastic MCA, ACA in G3 and G4 groups and normal MCA, ACA in G0 group were displayed in **Figure 5A**. The morphology of MCA and ACA in G1, G2 groups were similar to those in G0 group. The diameter of MCA and ACA in G3 and G4 groups at day 1 and 3 were similar, but smaller than those at day 5 and 7 (**Figure 5C** and **D**). The three dimensional micro-XCT images of normal (a) and SAH (b) rats were illustrated in **Figure 5E**. The vasospasm of MCA and ACA in a SAH rat could be obviously distinguished through the 3D micro-XCT photo (**Figure 5E** **b**).

## Discussion

In this study, we used SRA to evaluate cerebral vasospasm (CV) in rats with experimental SAH. CV is a major complication of SAH. It is still a challenge of diagnosis and therapy in humans. DSA, magnetic resonance angiography (MRA) and CTA are widely used to detect CV in humans. However, the relative low resolution of DSA, MRA and CTA limits the application in small animal models [8], [40]. Up to now, most of experimental SAH studies were based on indirect observation or pathological outcomes. Developing a novel technique, which can directly observe and measure vessel diameter changes in small animal models, is extremely important. SRA provides a unique tool for this purpose. In fact, it has been used in other SAH study [41]. In this study, we firstly applied synchrotron radiation angiography to directly observe, evaluate and record the course of cerebral vasospasm in living SAH animal models.

It was interesting that the results of cerebral vascular diameters between images of SRA and sections of histology were not exactly consistent with each other: MCA and ACA diameter measured in histological sections were much smaller than those measured in SRA images. Saline dehydration and paraformaldehyde fixation of brains after animal termination might result in values of diameter measured in histological sections being smaller than their actual values. As shown in **Figure 4**, MCA and ACA diameter of these two models at day 3 were the smallest; but the histological modality could not identify these tiny distinctions. Furthermore, some investigators did not detect CV of ACA in models of endovascular perforation and prechiasmatic cistern injection through measurement of histological sections 2 days after SAH [42]. Therefore, we presumed histological measurement was not as sensitive as SRA to detect fine pathological changes of cerebral vessels.

We applied micro-XCT to detect and evaluate cerebral artery changes in normal and SAH rats. We found that micro-XCT could distinct vasospastic changes in the Circle of Willis. The benefit of micro-XCT was that entire brain could be imaged with three dimensions (**Figure 5E**). However, the performance of micro-XCT imaging is relatively complicated and cannot be applied for monitoring vascular changes in living animals.

CBF measurement is regarded as a means to evaluate CV. Using ^14^C- iodoantipyrine or [^14^C]-IMP to measure rCBF, the lowest rCBF was detected at day 3–7, returned to normal after day 14 [28], [30], [43], [44]. In this study, we found rCBF in G3 and G4 groups decreased to the minimal value at 3 days of SAH and recovered to 50% at 7 days of SAH. Our results of [^125^I]-IMP was agree with the course vasospasm, which was observed by SRA. However, this CBF measurement only provided terminal evaluations.

Surface CBF measured by LDF was deemed as an *in vivo* method. The CBF of MCA supplying area began to decrease immediately after SAH induction and reached the lowest CBF only in a few seconds, and it maintained at a steady low level up to 150 minutes [45]. We drilled four holes to measure cortical CBF; we observed the lowest CBF at 30 minutes after SAH induction, which was in alignment with the results of Westermaier's. The changes of surface CBF at 1 to 7 days of SAH were corroborated with rCBF changes at 1, 3, 5 and 7 days of SAH, but they did not match each other entirely. The positive correlation of SRA imaging and surface CBF (**Figure 4E** and **F**) supported the hypothesis that diameter changes of cerebral artery reflected the tendency of CBF changes.

The limitation of SRA is that this performance is an invasive and irradiative technique: a PE-10 tube has to be inserted into ECA; therefore, the catheterized ECA had to be sacrificed. In addition, the imaging window is currently limited to 45 mm×2.5 mm, and it has to be scanned at least four frames to cover the whole hemispheric brain area. Finally, X-ray can damage brains. To limit the changes of blood vessels caused by radiation, we chose different groups of rats for SRA study after SAH. Using real time SRA to detect fine changes of cerebral vessels through accomplishment of intravenous cerebral angiography and low irradiation may be developed [46].

We chose two injection models to perform this study instead of artery puncture model owing to the operated simplicity and clinical similarity. To exclude the flaws of these two injection models, we made an additional group (n = 11) of cerebral artery puncture SAH model according to the previous report [25]. We found that the mortality in this model was 27%, which was similar with blood injection models. While neurological score in this model was higher, suggesting the clinical symptom of this vessel puncture model was not so severe as that of injection models (**Figure S2**).

There were many controversies of these two injection models. The advantage of prechiasmatic cistern injection model was its similarity to clinical rupture of aneurysm, but the mortality was high [27]. The mortality of cisterna magna injection model was low [28], [30]; however, whether this model could produce pronounced vasospasm of the circle of Willis, especially anterior circulation arteries, remained unfathomed.

In our study, mortality of cisterna magna injection model was 23%, which was consistent with some studies [47]–[49], but it was much higher than our expectation. According to the intracranial pressure (ICP) monitor (**Figure 2A** and **B**), the mortality was probably not due to high ICP into cisterna magna. We considered the high mortality of cistern magna injection SAH model was on account of the subarachnoid base, at which we injected blood, being close to the juncture of medulla and spinal cord. Furthermore, one rat of saline cisterna magna injection died during operation, we believed the death might be the result of brain stem injury from the PE-10 tube or the little bulb for ICP measurement. It suggested that the technique used to induce cisterna magna injection might possibly result in brain stem injury. Mortality of prechiasmatic cistern injection model was 13%, which was lower than Prunell's report [27] and in accordance with another study [50].

According to the results of LDF-CBF, [^125^I]-IMP CBF (including radio-autographic images, **Figure S1**), histological outcomes and SRA, the course of anterior circulation arteries vasospasm in these two models appeared similar. However, the diameter of ACA in SRA images suggested that the vasospasm resulting from prechiasmatic cistern injection was more pronounced. It may be due to blood volumes in basal subarachnoid [27]. At day 7 after SAH, vasospasm on the ACA in cisterna magna injection model was hardly distinguished. In contrast, vasospasm on anterior circulation arteries in prechiasmatic cistern injection model was still identifiable. It implied that the model made by prechiasmatic cistern injection could produce more pronounced and lasting vasospasm of anterior circulation arteries.

In summary, SRA provides a practical and precise tool, which can directly evaluate cerebral vasospasm in small animal experimental subarachnoid hemorrhage models. The vasospasm courses of these two injection models are similar; however, model produced by prechiasmatic cistern injection is more suitable for study of anterior circulation vasospasm.

## Supporting Information

Figure S1 **The BAS-MS type of imaging plate and the BAS-2500 imaging reader (Fuji Film Co., Tokyo, Japan) were used in the radio-autographic study.** These ^125^I-labeled brain samples, as well as 6 reference ^125^I samples, were directly contacted with the imaging plate in a lead shielding cassette for 24 hours. After the irradiation, the images were read out and analyzed using the software Multi Gauge version 3.1 (Fuji Film Co., Tokey, Japan). The images are from normal control group (a), cisterna magna injection SAH group (b) and prechiasmatic cistern injection SAH group (c).
(TIF)

Figure S2 **Bar graphs display neurobehavioral scores of cisterna magna injection SAH group (CM), prechiasmatic cistern injection SAH group (PC) and cerebral artery perforation SAH group (puncture).** Data are means±SD, n = 8.
(TIF)
