# Supplementary figures and images for: A Novel Intravital Method to Evaluate Cerebral Vasospasm in Rat Models of Subarachnoid Hemorrhage: A Study with Synchrotron Radiation Angiography
Source: PLoS One. 2012 Mar 12;7(3):e33366. doi: 10.1371/journal.pone.0033366 (PMC3299776; doi:10.1371/journal.pone.0033366)

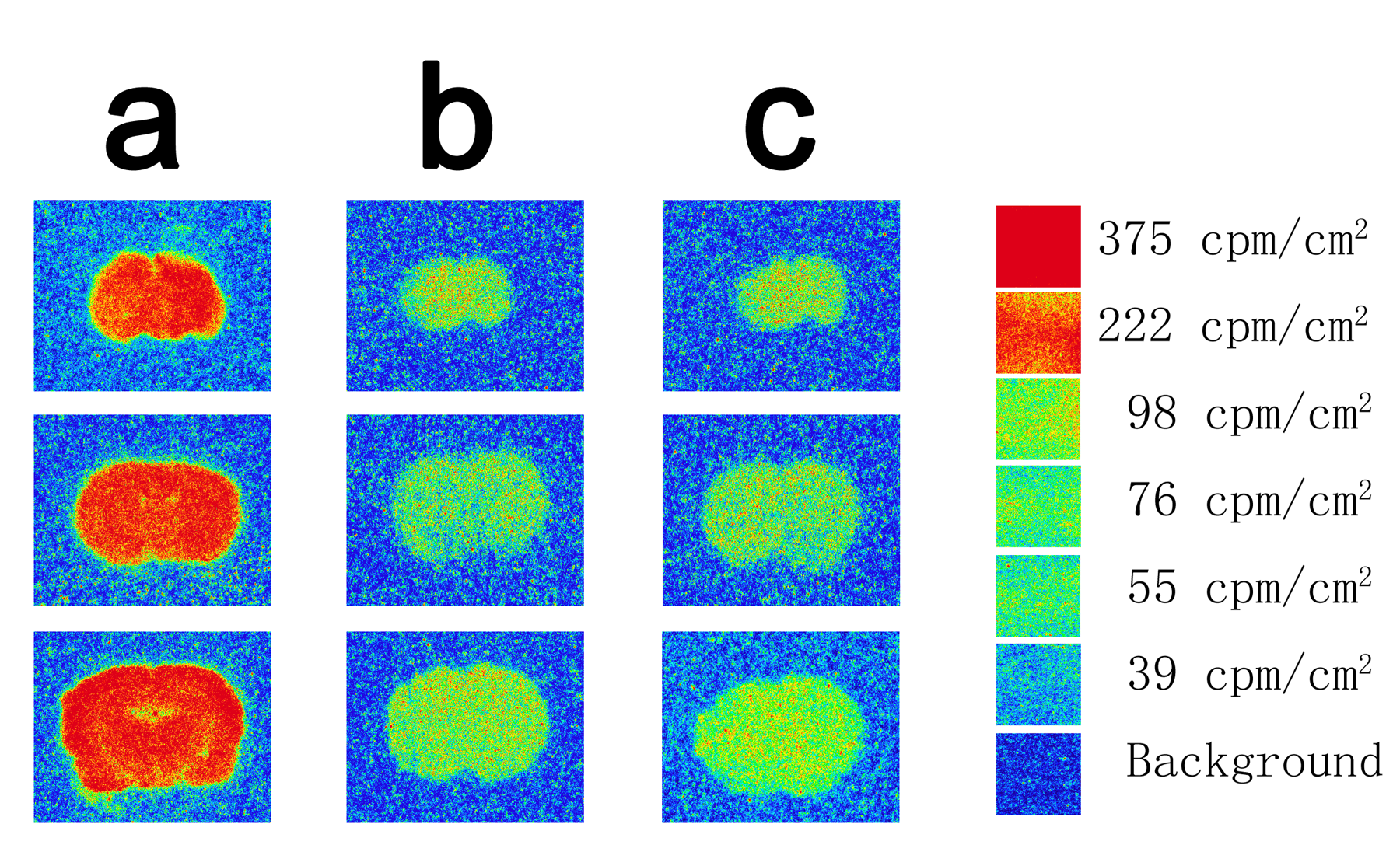

Supplement: Figure S1 — The BAS-MS type of imaging plate and the BAS-2500 imaging reader (Fuji Film Co., Tokyo, Japan) were used in the radio-autographic study. These 125I-labeled brain samples, as well as 6 reference 125I samples, were directly contacted with the imaging plate in a lead shielding cassette for 24 hours. After the irradiation, the images were read out and analyzed using the software Multi Gauge version 3.1 (Fuji Film Co., Tokey, Japan). The images are from normal control group (a), cisterna magna injection SAH group (b) and prechiasmatic cistern injection SAH group (c). (TIF) [file pone.0033366.s001.tif]

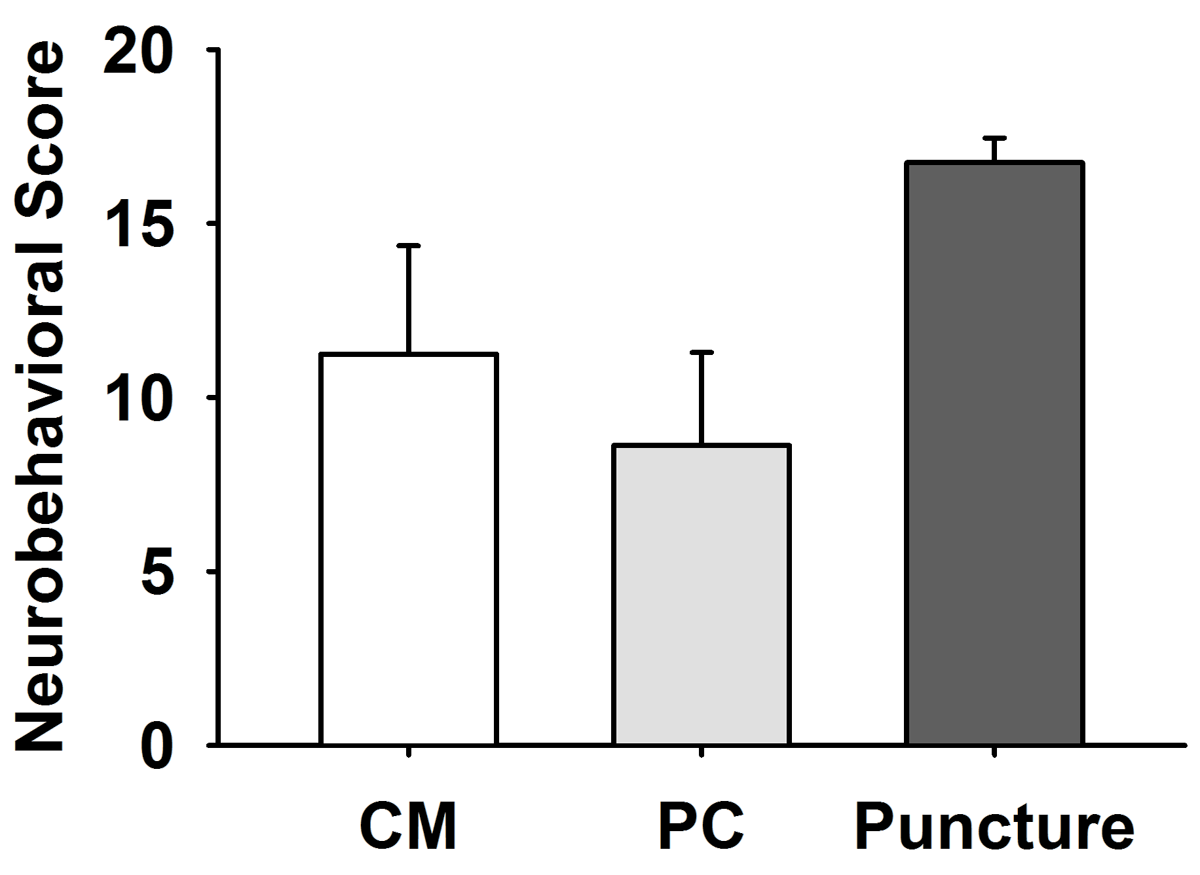

Supplement: Figure S2 — Bar graphs display neurobehavioral scores of cisterna magna injection SAH group (CM), prechiasmatic cistern injection SAH group (PC) and cerebral artery perforation SAH group (puncture). Data are means±SD, n = 8. (TIF) [file pone.0033366.s002.tif]
